# Interventricular Differences in Action Potential Duration Restitution Contribute to Dissimilar Ventricular Rhythms in *ex vivo* Perfused Hearts

**DOI:** 10.3389/fcvm.2019.00034

**Published:** 2019-04-03

**Authors:** Balvinder S. Handa, Saheed Lawal, Ian J. Wright, Xinyang Li, Javier Cabello-García, Catherine Mansfield, Rasheda A. Chowdhury, Nicholas S. Peters, Fu Siong Ng

**Affiliations:** ^1^National Heart and Lung Institute, Imperial College London, London, United Kingdom; ^2^Imperial College Healthcare NHS Trust, London, United Kingdom

**Keywords:** ventricular tachycardia, ventricular fibrillation, implantable cardioverter-defibrillator, dissimilar rhythms, dissimilar ventricular rhythms, action potential duration restitution, lidocaine, antiarrhythmic drugs

## Abstract

**Background:** Dissimilar ventricular rhythms refer to the occurrence of different ventricular tachyarrhythmias in the right and left ventricles or different rates of the same tachyarrhythmia in the two ventricles.

**Objective:** We investigated the inducibility of dissimilar ventricular rhythms, their underlying mechanisms, and the impact of anti-arrhythmic drugs (lidocaine and amiodarone) on their occurrence.

**Methods:** Ventricular tachyarrhythmias were induced with burst pacing in 28 Langendorff-perfused Sprague Dawley rat hearts (14 control, 8 lidocaine, 6 amiodarone) and bipolar electrograms recorded from the right and left ventricles. Fourteen (6 control, 4 lidocaine, 4 amiodarone) further hearts underwent optical mapping of transmembrane voltage to study interventricular electrophysiological differences and mechanisms of dissimilar rhythms.

**Results:** In control hearts, dissimilar ventricular rhythms developed in 8/14 hearts (57%). In lidocaine treated hearts, there was a lower cycle length threshold for developing dissimilar rhythms, with 8/8 (100%) hearts developing dissimilar rhythms in comparison to 0/6 in the amiodarone group. Dissimilar ventricular tachycardia (VT) rates occurred at longer cycle lengths with lidocaine vs. control (57.1 ± 7.9 vs. 36.6 ± 8.4 ms, *p* < 0.001). The ratio of LV:RV VT rate was greater in the lidocaine group than control (1.91 ± 0.30 vs. 1.76 ± 0.36, *p* < 0.001). The gradient of the action potential duration (APD) restitution curve was shallower in the RV compared with LV (Control - LV: 0.12 ± 0.03 vs RV: 0.002 ± 0.03, *p* = 0.015), leading to LV-to-RV conduction block during VT.

**Conclusion:** Interventricular differences in APD restitution properties likely contribute to the occurrence of dissimilar rhythms. Sodium channel blockade with lidocaine increases the likelihood of dissimilar ventricular rhythms.

## Introduction

Dissimilar intracardiac rhythms refer to the occurrence of different tachyarrhythmias or different rates of the same tachyarrhythmia between the right and left ventricles or the right and left atria. Dissimilar atrial rhythms were first described in the atria in the early nineteenth century (1, 2). A study by Zipes established proof for existence of dissimilar atrial rhythms by inducing and documenting simultaneous atrial fibrillation in one atrium and a slower regularized rhythm in the contralateral atrium in a case series of 10 patients (3). Since then, the presence of dissimilar atrial rhythms has been widely documented and confirmed (3–5). However, much less is known about dissimilar rhythms in the ventricles.

Dissimilar ventricular rhythms were first reported in patients undergoing cardiac surgery on cardiopulmonary bypass, whereby in some patients sinus rhythm was recorded in the right ventricle (RV) with concomitant ventricular fibrillation (VF) in the left ventricle (LV) (6). Dissimilar ventricular rhythms, whilst rare, have since also been recorded in patients with implantable cardiac resynchronization therapy (CRT) devices. Concurrent continuous recording of right ventricle (RV) and left ventricle (LV) electrograms has only been possible in patients with CRT devices. Barold et al. performed a systematic analysis of 723 CRT-D devices returned for disposal and found 16 cases of dissimilar interventricular rhythms (7). Frequently, during episodes of ventricular tachycardia (VT), they found that the rate recorded in the RV was slower than the LV. Instances of 2:1 LV to RV conduction, and VT and ventricular fibrillation (VF) existing simultaneously in different ventricles was reported. The slower VT rate in the RV may have the consequence of defibrillators inappropriately withholding therapy as the rates measured in the RV fall below the threshold for ICD therapy. Whilst dissimilar ventricular rhythms have been clinically observed and reported, there is no explanation for the underlying mechanism by which they occur. Intraventricular conduction block has been postulated as a possible mechanism (6, 8). The RV and LV have been shown to differ in their APD restitution response and ionic currents (9) and this may offer some explanation for the mechanism underlying dissimilar ventricular rhythms.

In this study, we set out to investigate the inducibility of dissimilar ventricular rhythms and the condition under which they occur. We further investigated the impact of commonly used antiarrhythmics, including lidocaine, a class Ib antiarrhythmic, and amiodarone, a class III agent, on the threshold for developing dissimilar ventricular rhythms, and utilized their differing impact on ionic currents to probe the underlying mechanism using optical mapping.

## Methods

### Experimental Protocols

#### Induction of Dissimilar Ventricular Rhythms

Twenty eight Sprague-Dawley (SD) rats (250–300 g) were euthanized and the hearts explanted, heparinized, and rapidly perfused *ex-vivo* on a Langendorff apparatus with Krebs-Henseleit solution (in mmol/l: NaCl 118.5, CaCl_2_ 1.85, KCl 4.5, glucose 11.1, NaHCO_3_ 25, MgSO_4_ 2.5, NaH_2_PO_4_ 1.4) gassed with 95% O_2_/5% CO_2_ at 37 ± 0.5°C and pH 7.35 ± 0.05. A 10 min stabilization period was allowed during which the flow rate (10–15 ml/min), temperature (37 ± 0.5°C) and perfusion pressure through the aorta was maintained between 90 and 100 mmHg.

Programmed electrical stimulation was carried out with silver electrodes placed at the bases of the left and right ventricles with a MicroPace system (Micropace EP, Santa Ana, USA). A burst pacing protocol (2 mA, cycle length 50–70 ms, 30 beat train) was used to induce ventricular tachyarrhythmias. A 10–30 s period of global ischaemia followed by reperfusion was used in combination with burst pacing protocols if the ventricular tachyarrhythmia could not be initiated or sustained. The hearts received control perfusate (*n* = 14), with perfusate containing 10 μM lidocaine (*n* = 8) or perfusate containing 10 μM amiodarone (*n* = 6). Lidocaine and amiodarone were pre-infused for 15 min before arrhythmia induction protocols.

Electrograms were recorded from the RV lateral free wall and LV anterolateral wall simultaneously using Labchart 7.0 (AD Instruments, Sydney, Australia) with a custom made bipolar silver electrodes. Electrodes were connected to a Bioamplifier and a PowerLab data acquisition system (AD Instruments, Sydney, Australia). The electrograms were continuously tracked to document dissimilar ventricular rhythms. Dissimilar rhythms were defined as either interventricular VT and VF co-existing, or dissimilar interventricular rates of either VT or VF lasting more than 30 s.

### Optical Mapping of Dissimilar Ventricular Rhythms

In order to study the underlying mechanisms of dissimilar ventricular rhythms, a further 14 SD rats (250–300 g) hearts were explanted and Langendorff perfused as above with Krebs-Henseleit solution (6 control, 4 amiodarone, 4 lidocaine). Optical mapping fluorescence data were recorded respectively from the LV and RV epicardial surface with simultaneous recording of biventricular electrograms. The transmembrane voltage was recorded with optical mapping using our custom made complementary metal oxide semiconductor (CMOS) camera (Cairns, Feversham UK) utilizing the potentiometric dye RH237 (25 μl of 1 mg/ml DMSO; Thermo-Fisher, Massachusetts, USA) and excitation contraction uncoupler blebbistatin (10 μmol/L, Tocris Bio-Sciences, Cambridge UK). All our methods for Langendorff-perfusion and optical mapping analysis have been previously described in detail (10, 11). Dissimilar ventricular rhythms were induced with the same burst pacing protocol and the mechanism optically mapped.

### Data Analysis and Statistics

Optical mapping data were analyzed and APD calculated from the transmembrane voltage as previously described (10–13) using MATLAB R2017a software (MathWorks, Marlborough, Massachusetts) and the open source RHYTHM MATLAB script (12). Phase analysis of VF data was performed using our custom MATLAB script, as previously described (14, 15).

For comparison between groups, the data's fit to a normal distribution was assessed with a Q-Q plot. *T*-tests were used to compare means between two groups. Where three or more groups were compared, 1-way ANOVA with a *post-hoc* Bonferroni's multiple comparison test was used. RV and LV restitution curves were analyzed utilizing the linear regression method. Results were considered statistically significant with a *p*-value of < 0.05. All values are mean ± SEM.

## Results

### Clinical Occurrence of Dissimilar Ventricular Rhythms Recorded on a CRT-D Device

**Figure 1:**
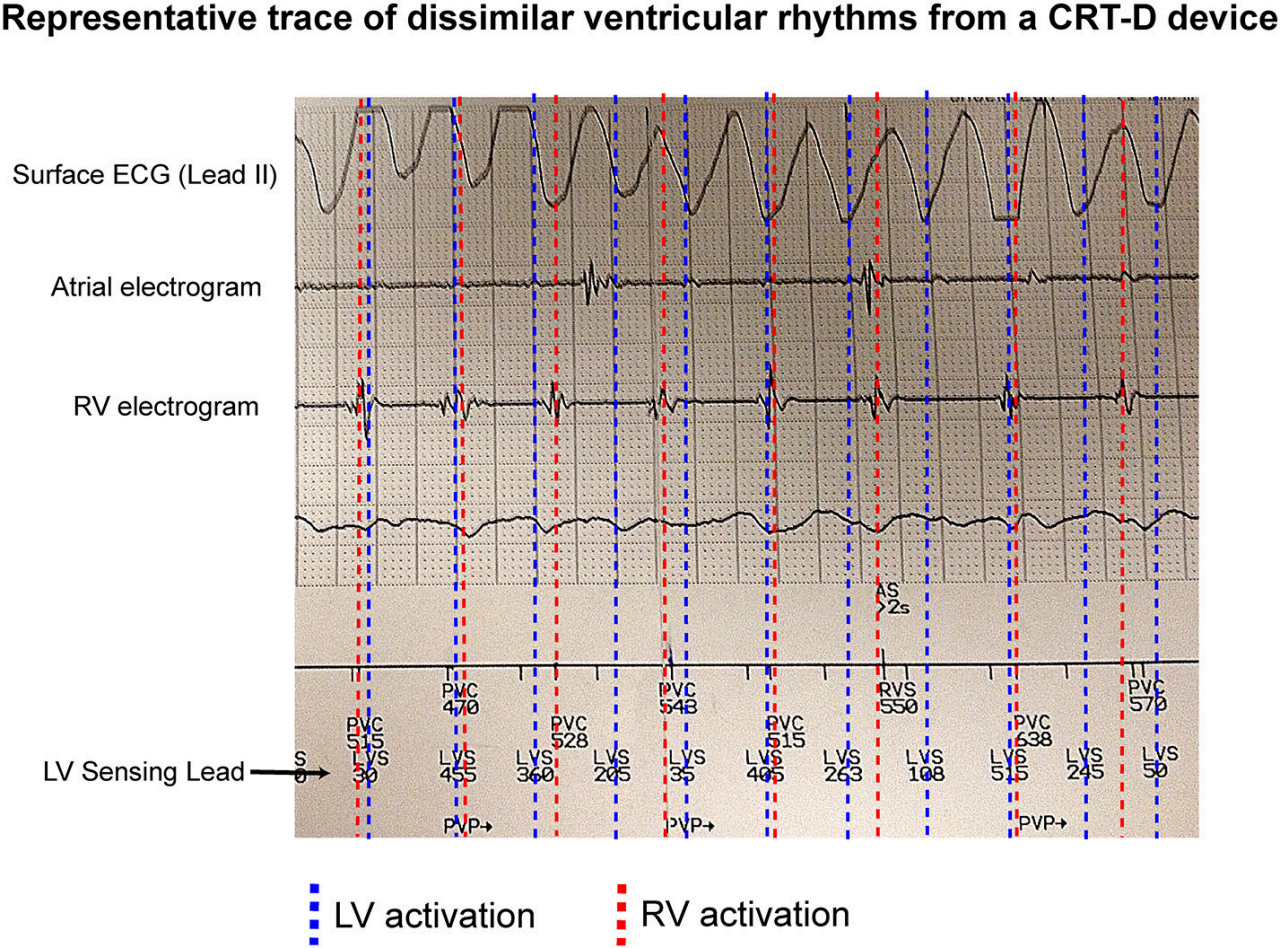
Dissimilar ventricular rhythms recorded on a CRTD. An example of a CRT-D trace showing a patient with VF recorded on a surface ECG, and a regular slow RV activation rate compared to a dissimilar irregular LV activation rate (recorded as LVS) from the LV sensing lead.

Figure 1 shows an example of an occurrence of dissimilar ventricular rhythms in a patient with a CRT-D device *in situ*. This patient was recorded having VF on the surface ECG. The RV electrogram shows a regular rhythm in keeping with VT, which is running both dyssynchronous and at a slower rate to an irregular LV rhythm, which is likely VF.

### Dissimilar Rhythms Are Inducible and Dissimilar Rates Occur More Frequently in VT Than VF

**Figure 2:**
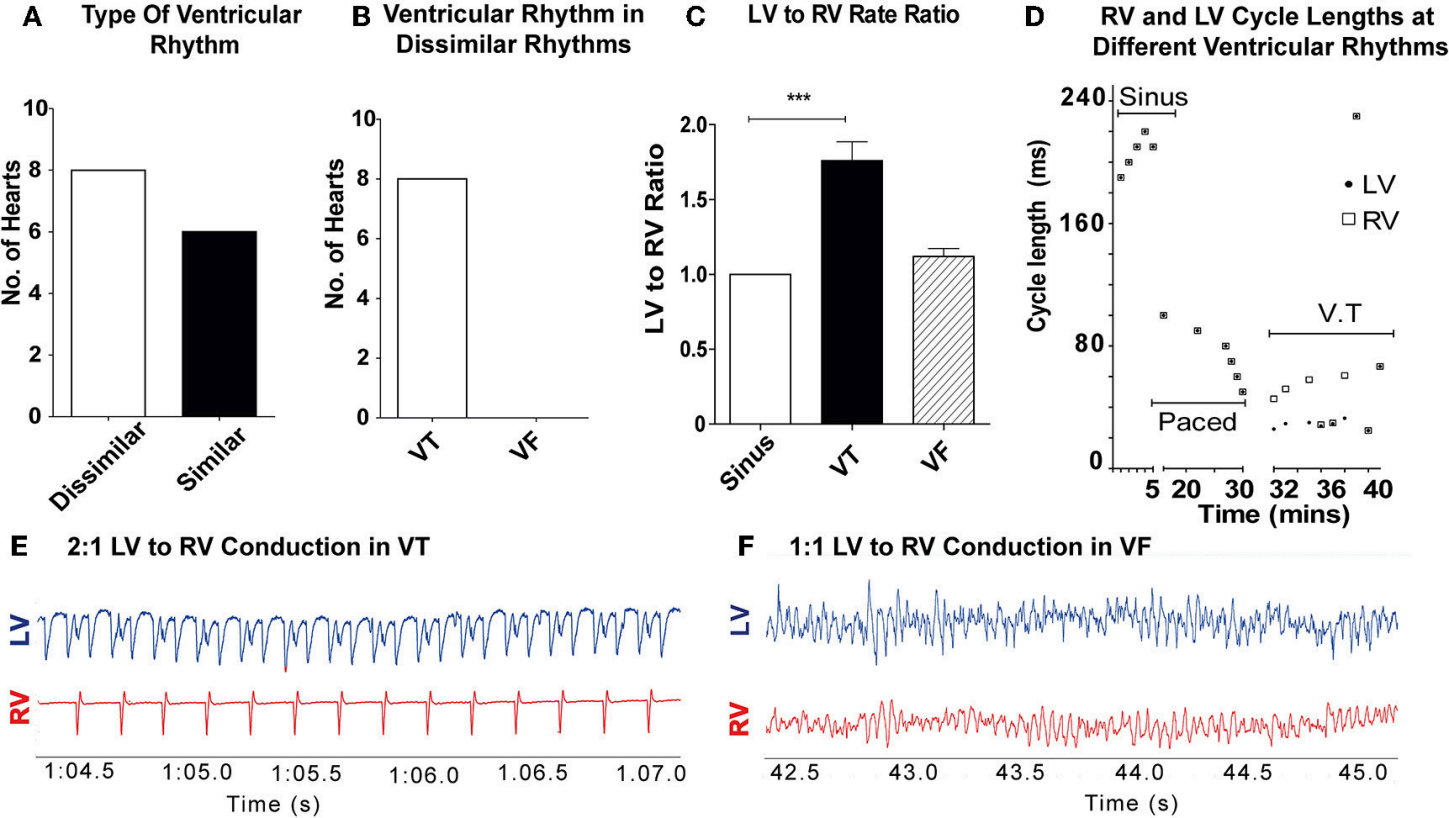
Dissimilar rhythms are inducible in *ex-vivo* perfused hearts: **(A)** Type of ventricular rhythm induced in control hearts **(B)** Type of dissimilar rhythm induced **(C)** Ratio of LV to RV rate in VT and VF **(D)** Representative graph of a single experiment showing CL of RV and LV conduction in different ventricular rhythms with dissimilar VT CLs. **(E)** Representative electrogram trace for a single heart in VT showing 2:1 LV to RV rate **(F)** and 1–1 LV to RV rate in VF. LV, left ventricle; RV, right ventricle; CL, cycle length(s); SR, sinus rhythm; VT, ventricular tachycardia; VF, ventricular fibrillation [Data from control hearts (*n* = 14), ***p < 0.001, 1-way ANOVA, *post-hoc* Bonferroni test].

11/28 hearts sustained a ventricular tachyarrhythmia with burst pacing alone, 17/28 hearts (8 controls, 5 amiodarone, and 4 lidocaine) required ischaemia-reperfusion in addition to burst pacing to sustain a ventricular tachyarrhythmia. In the control hearts (*n* = 14), dissimilar rhythms were induced in 8/14 hearts (57%; Figure 2A). Of these, all were VT with dissimilar rates between ventricles (Figure 2B). In the remaining 6/14 (43%), only sustained VF with similar rates between ventricles were observed. When VT was induced, most commonly 2:1 LV to RV rates occurred (Figure 2E). The VT rate in the RV was slower than the LV, with a LV to RV rate ratio of 1.76 ± 0.36 in VT and 1 ± 0 in sinus rhythm (SR) (*p* < 0.001). During VF, there was no significant difference in LV to RV rates in comparison to SR (Figures 2C,F). When dissimilar rhythms developed, the cycle length of the tachyarrhythmia and the ratio of LV to RV rate showed small fluctuations over the recording period (Figure 2D).

### Lidocaine Lowers the Threshold at Which Dissimilar Rhythms Develop

**Figure 3:**
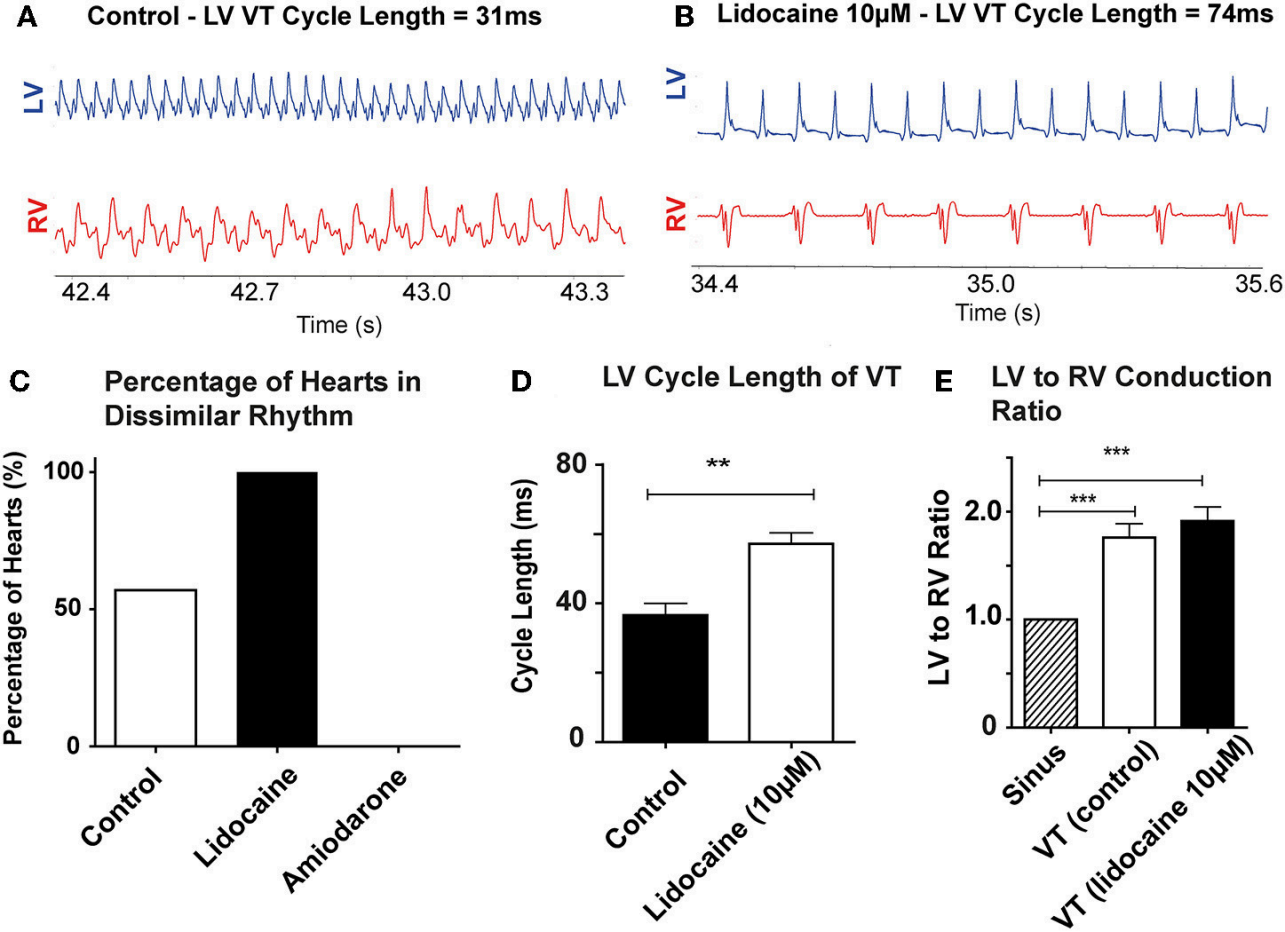
Lidocaine lowers threshold for dissimilar VT rates: Representative bipolar electrograms of LV and RV in VT in **(A)** a control heart **(B)** a heart perfused with 10 μM Lidocaine showing dissimilar VT rates occurring at longer LV CLs with lidocaine. **(C)** Increased propensity to develop dissimilar rhythms with 10 μM lidocaine compared to control and 10 μM amiodarone treated hearts **(D)** LV CL of VT is longer with lidocaine 10 μM **(E)** LV to RV conduction ratio in VT with dissimilar interventricular rates is higher in control and lidocaine 10 μM perfused hearts. [Data from control (*n* = 14), 10 μM lidocaine (*n* = 8), and 10 μM amiodarone (*n* = 6) hearts, **p < 0.01, ***p < 0.001, *t*-test **(D)**, and 1-way ANOVA with *post-hoc* Bonferroni **(E)**]. Abbreviations as per Figure 2.

Of the hearts in the antiarrhythmic drugs group, pre-treatment with 10 μM lidocaine (*n* = 8) reduced the threshold at which dissimilar ventricular rhythms developed. In the lidocaine group 8/8 hearts (100%) developed dissimilar rhythms during the recording period, and of these all were VTs with dissimilar rates. Pre-treatment with 10 μM amiodarone (*n* = 6) reduced the occurrence of dissimilar rhythms, and only VF with similar rates was observed in this group (Figure 3C).

Lidocaine prolonged the cycle length of VT in the LV when dissimilar VT rates were recorded (control: 36.6 ± 8.4 vs. lidocaine: 57.1 ± 7.9 ms, *p* < 0.001; Figure 3D). The RV rate always remained slower than the LV (Figures 3A,B). The ratio of LV to RV rate in these hearts in VT was increased compared to SR in both groups; SR vs. VT (control): 1 ± 0 vs. 1.76 ± 0.36 (*p* < 0.001) and SR vs. lidocaine 10 μM: 1 ± 0 vs. 1.91 ± 0.30 (*p* < 0.001; Figure 3E). Whilst there was trend toward increased ratio of LV to RV rate in VT with lidocaine, there was no significant difference between VT groups.

### The RV and LV Differ in Their APD Restitution Properties

**Figure 4:**
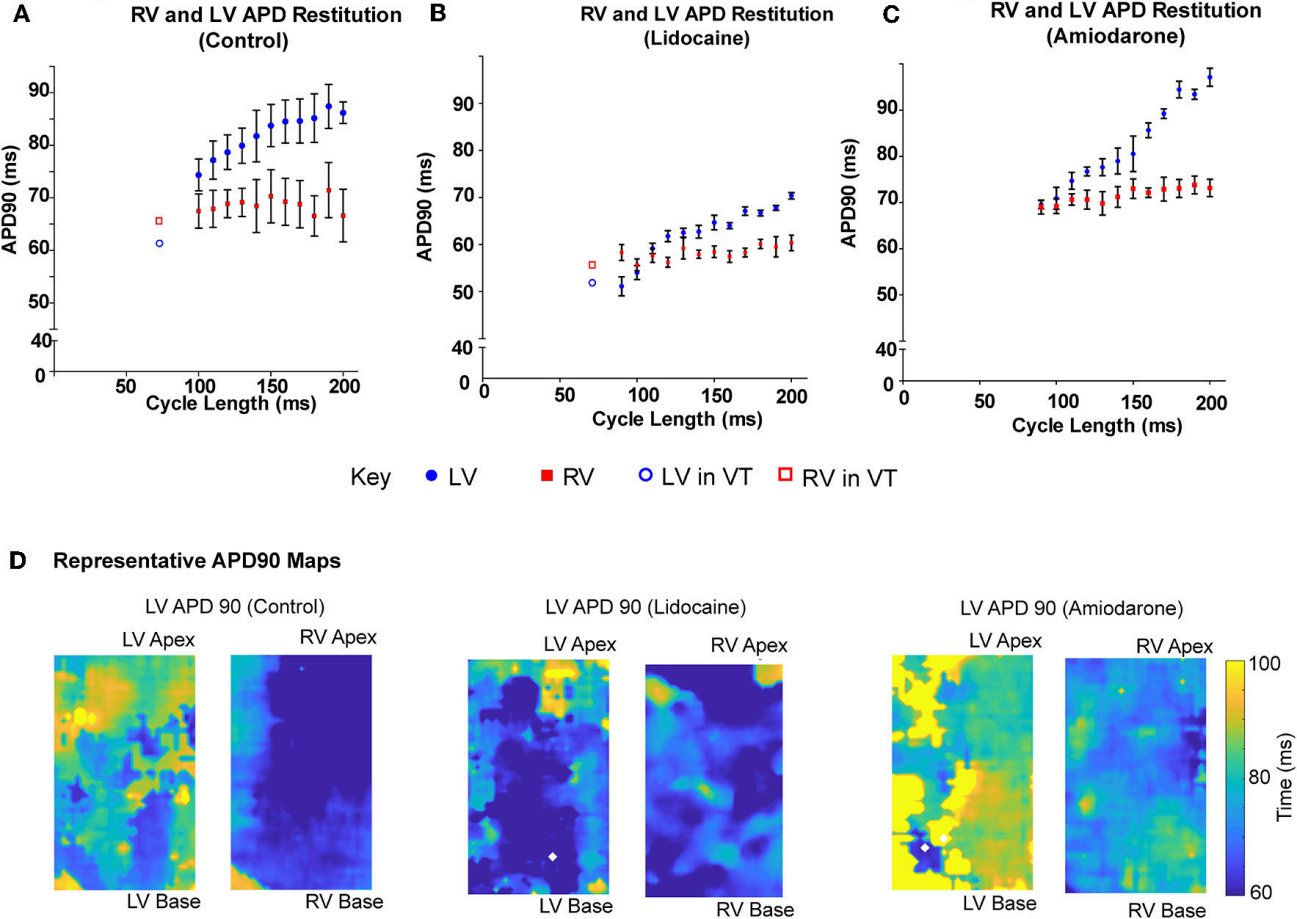
LV APD shortens more than RV at shorter pacing cycle lengths. LV and RV APD restitution curves at different paced cycle lengths showing that the LV APD restitution curve has a steeper gradient than the RV APD restitution curves in **(A)** control, **(B)** lidocaine, and **(C)** amiodarone hearts. □/° showing LV and RV APD in sample VT data set with dissimilar rhythms. **(D)** Representative APD90 maps from control (left), lidocaine (middle), and amiodarone (right) hearts. [Data from control (*n* = 6), lidocaine (*n* = 4), and amiodarone (*n* = 4) hearts, linear regression].

When paced at decrementing cycle lengths, the LV APD90 restitution gradient is steeper than the RV (0.12 ± 0.03 vs. 0.002 ± 0.03, *p* = 0.015; Figure 4A). Where we optically mapped VT with dissimilar rates in a single data set, with a cycle length shorter than we could capture through external pacing, the LV APD90 continued to shorten, whereas the RV plateaued, remaining relatively unchanged (Figure 4A). In the lidocaine group, the LV APD90 restitution gradient was steeper than the RV (0.15 ± 0.01 vs. 0.02, *p* < 0.0001). At very short pacing cycle length of 90 and 100 ms, and in dissimilar interventricular VT rates, the LV APD90 was shorter in the LV than the RV (Figure 4B). Similarly, in the amiodarone group, the LV APD90 restitution gradient was steeper than the RV (0.26 ± 0.02 vs. 0.04 ± 0.01, *p* < 0.0001). However, the amiodarone LV restitution slope gradient was much steeper than both the control and lidocaine group (Amiodarone – 0.26 ± 0.02, Lidocaine – 0.15 ± 0.01, Control – 0.12 ± 0.03, *p* < 0.001, Figure 4C). APD90 was shortened in the lidocaine group and prolonged in the amiodarone group in both the LV and RV. There was a great degree of the APD90 heterogeneity in the amiodarone group comparative to the control and lidocaine group (Figure 4D).

### Mechanisms of Dissimilar Rhythms

Of the control hearts that were optically mapped, dissimilar rhythms were induced in 3/14 heart (2 control, 1 lidocaine). The mechanism of dissimilar VT rates and dissimilar ventricular tachyarrhythmias (VT and VF) were recorded.

### Interventricular Conduction Block Occurs in Dissimilar VT Rates

**Figure 5:**
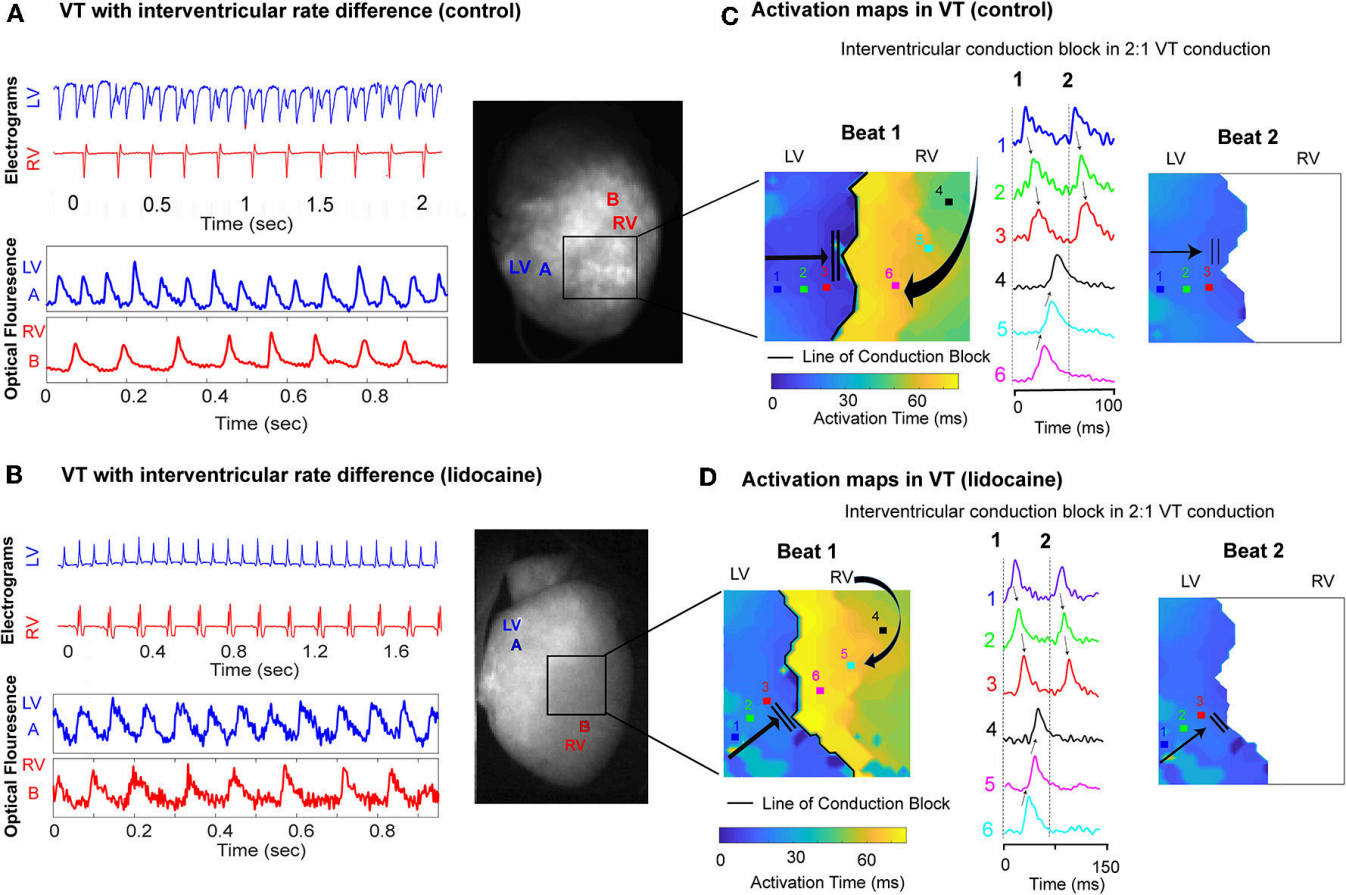
Interventricular conduction block in dissimilar ventricular VT rates. Representative LV and RV bipolar electrograms (top), optical mapping signals (bottom) with corresponding sampling sites (right) in **(A)** control and **(B)** lidocaine perfused hearts showing interventricular rate differences in VT. Corresponding activation maps and optical fluorescence data showing interventricular conduction block as the mechanism of 2:1 LV to RV VT rate in control **(C)** and lidocaine **(D)** perfused hearts.

In dissimilar VT rates we observed a 2:1 ratio of LV to RV conduction on the bipolar electrograms, with a much slower rate in the RV. Simultaneous optical mapping showed a line of interventricular conduction block as the likely mechanism in both the control and lidocaine group (Figures 5A,B). Activation maps in VT showed a well-defined line of conduction block in both groups, and the first propagating wavefront was blocked between the ventricles and had to take a longer path to be conducted from LV to RV. The wavefront that followed was blocked and not conducted from LV to RV (Figures 5C,D).

### Dissimilar Rhythms With Co-existing VF and VT

**Figure 6:**
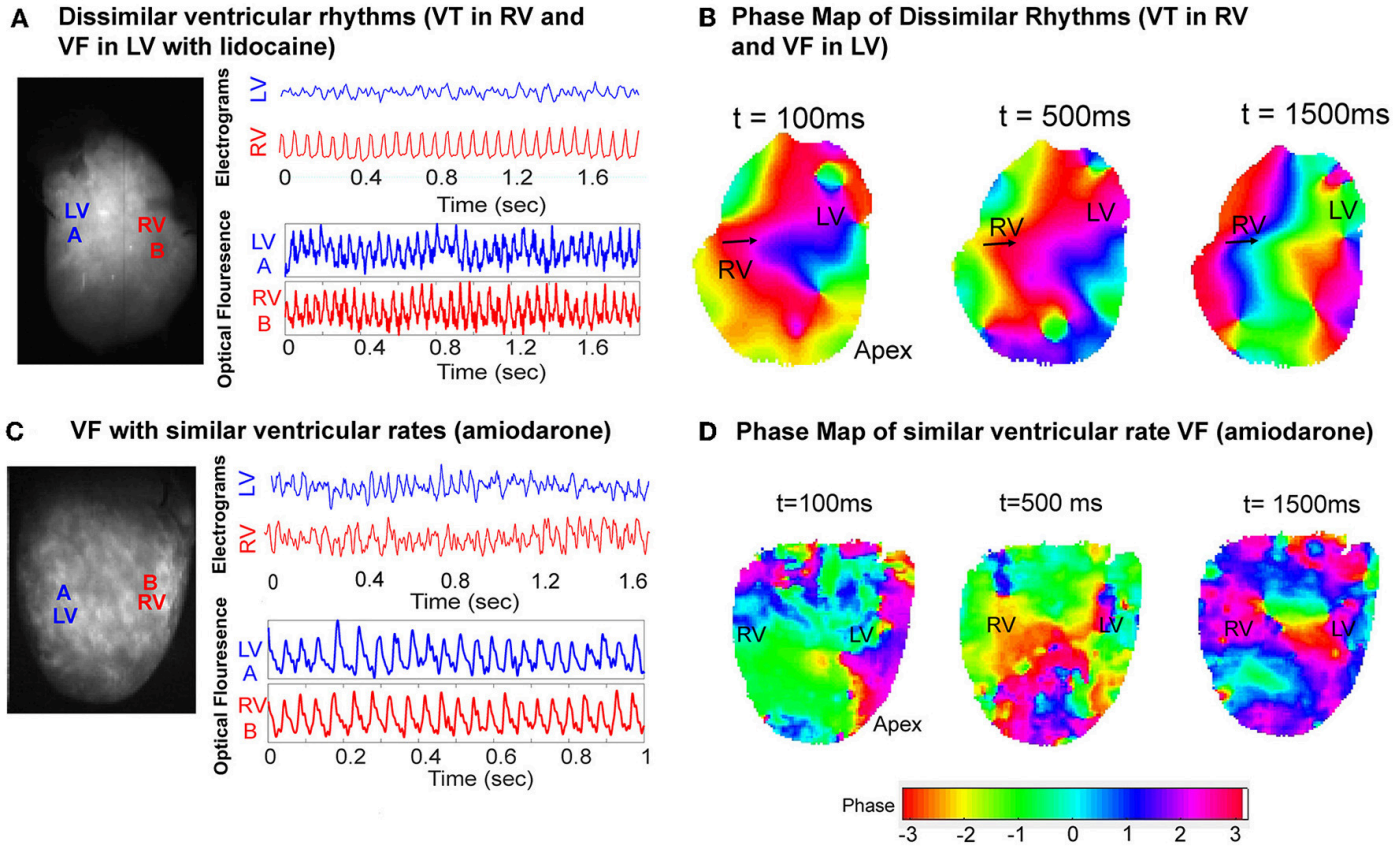
VF phase maps. **(A)** Representative LV and RV bipolar electrograms (top) and optical mapping signals (bottom) with corresponding sampling sites (left) in a control heart showing dissimilar ventricular tachyarrhythmias (VT and VF). **(B)** Corresponding VF phase map of **(A)** showing regular periodic RV activation and contrasting more disorganized LV activation (Supplementary Data–Video 1). **(C)** Representative LV and RV bipolar electrograms (top) and optical mapping signals (bottom) with corresponding sampling sites (left) in an amiodarone perfused heart, and the corresponding VF phase map **(D)** showing disorganized VF with similar rates.

Dissimilar ventricular tachyarrhythmias, where VF and VT occurred simultaneously in the left and right ventricles, were also optically mapped. Figure 6 shows one instance of this phenomenon in a representative control heart. The ventricular bipolar electrograms show simultaneous VF in the LV and VT in the RV (Figure 6A). Optical mapping data recorded synchronously, processed with phase analysis showed differing patterns of activation between the RV and LV. There is a regular wavefront that activated RV from the lateral wall to the septum causing a monomorphic VT in the RV. However, the activation in the LV shows a more disorganized activation pattern (Figure 6B, Supplementary Data–Video 1). In the amiodarone group we only observed chaotic VF with similar ventricular rates (Figures 6C,D).

## Discussion

In this study, we demonstrated that dissimilar interventricular rhythms are inducible and that interventricular rate differences occur more frequently during VT than VF. VT with slower RV rates in comparison to the LV occurred frequently in our *ex-vivo* perfused rat heart preparation. Sodium channel blockade with lidocaine lowered the threshold at which dissimilar VT rates were observed. Optical mapping studies suggested that interventricular differences in APD restitution is a possible cause of dissimilar ventricular rhythms. We also demonstrated two possible mechanisms using optical mapping by which dissimilar VT rates and dissimilar ventricular tachyarrhythmias can occur, with both interventricular conduction block and disorganization of propagating wavefronts recorded.

### The RV and LV Differ in Their Embryological Development and Transcription Profiles

These observation of dissimilar rhythms may be explained by the interventricular electrophysiological phenotype differences. LV and RV differ significantly in structure, function and electrophysiology. Embryonically, the LV develops from the first heart field and RV matures from a separate progenitor population in the second heart field (16). During maturation cardiomyocytes demonstrate differing interventricular transcriptional profiles, which over time, become similar as they mature. However, transcriptional profiles can change under stress (17). Sivagangabalan et al. perfused myopathic human hearts *ex-vivo* from patients undergoing transplant surgery, induced ventricular fibrillation, and demonstrated differing ion channel gene expression in more than 20 genes in the RV and LV in response to VF (9). These intraventricular differences ion channel expression may influence ionic currents and explain the APD restitution differences we observed between the LV and RV in our study.

### Clinical Reports of Dissimilar Ventricular Rhythms

Clinically, the phenomenon of dissimilar ventricular rhythms whilst rare, has been observed and documented. A recent case report by Kubickova et al. highlights the potential serious clinical implications of dissimilar ventricular rhythms and ventricular rates. During VF sensitivity checks in an implantable cardioverter defibrillator (ICD) implant, the device failed to shock a patient in VF due to a slower concomitant RV VT below the treatment threshold zone (18). In this study, we were able to elicit dissimilar rates in VT and showed that in keeping with this case report, the RV rate was always slower. There are reports of ventricular fibrillation (VF) occurring in right ventricle (RV) simultaneously with SR in left ventricular (LV) and a case of this phenomenon was reported during ventricular disarticulation in a patient with right ventricular dysplasia (19). Simultaneous SR in the RV with VF in the LV has also been reported during cardiopulmonary bypass (6). We documented this rare occurrence of dissimilar ventricular rhythms with concomitant VT and VF in our *ex-vivo* perfused rat hearts.

### Interventricular Action Potential Duration Restitution Differences Contribute to Dissimilar Rhythms

APD restitution refers to adaptation of depolarization and repolarization to a changing heart rate. At faster heart rates, repolarization speeds up and APD effectively shortens. APD restitution is complex on a cellular level and changes in the interplay between a number of ionic currents have been described in response to faster heart rates. Briefly, as the heart rate increases the low steady state levels of intracellular calcium cannot be maintained and this activates the sodium-calcium exchanger (NCX) mediated calcium extrusion and sodium entry. The increased intracellular sodium is then rectified through enhanced activity of the sodium-potassium exchange*r* (I_NaK_) and an increase in the outward potassium currents (I_Ks_ and I_Kr_) follows, and this ultimately shortens the APD (20–22). The relationship by which APD changes depending on the heart rate is defined by the restitution curve.

Several studies have demonstrated that the propensity to ventricular arrhythmias can vary dependent on the slope of the APD restitution curve. A shallow or flat APD restitution curve can reduce vulnerability to VF by programmed electrical stimulation in a pig model (23). In simulation studies using human ventricular cell models, steep restitution slopes have been shown to promote re-entrant waves and VF by introducing repolarization alternans (24). In humans, APD restitution heterogeneity has been shown to promote cardiac electrical instability (25). In our study, we found the LV APD restitution curve was steeper than the RV, it is likely that this increases arrhythmogenic propensity of LV by allowing fast rate ventricular tachyarrhythmias to develop and perpetuate.

Whilst the phenomenon of dissimilar ventricular rhythms has been observed clinically, the mechanism(s) responsible remain undefined. We showed that LV and RV APD restitution curves vary significantly. Whilst LV APD can shorten effectively at decrementing pacing cycle lengths, the ability of the RV APD to do so is limited owing to its shallow restitution curve. This has been shown in a canine heart model where the RV APD responds differently to the LV in response to pacing, demonstrating comparatively reduced APD shortening and a shallower restitution curve with higher pacing rates (26). A recent study by Shattock et al. showed that interventions that prolong the APD result in steeper APD restitution slope gradients (27). In our experiments, the LV APD was significantly longer than RV and this may explain the steeper LV APD restitution gradient. In addition, we observed that amiodarone both prolonged the APD and increased the LV APD restitution gradient.

In keeping with our findings, in a study of VT ablation procedures in human subjects without structural heart disease, the slope of the LV restitution curve was found to be significantly steeper than the RV (28). In another study in patients undergoing cardiac surgery on cardiopulmonary bypass, a 256 electrode epicardial sock demonstrated a steeper LV restitution curve in comparison to the RV in addition to widespread regional differences within the same ventricle (29).

### Lidocaine Lowers the Threshold for Induction of Dissimilar Ventricular Rhythms

Lidocaine, commonly used to treat acute ventricular tachycardia, altered the threshold at which dissimilar VT rates were observed in our study, with dissimilar VT rates occurring more frequently and at longer cycle lengths. Lidocaine is a moderately potent class 1b antiarrhythmic agent that shortens APD and reduces the excitability of myocardial cells and slows conduction velocity through sodium channel blockade (30). In our study, it is likely VT was stabilized with this slowing of conduction velocity, and as such dissimilar VT rates became more likely. In the conventional model of anatomical re-entry, a slower conduction velocity would reduce the likelihood of wavefront interaction with a refractory tail and stabilize VT by allowing it to perpetuate. Sodium channel blockade itself may have differential effects on the ventricles. Cardiac sodium channels show heterogeneity in expression transmurally in the ventricular wall and across cardiac chambers (31–33). Thus, augmentation of sodium ionic currents by pharmacological agents like lidocaine could cause inhomogeneous conduction velocity slowing and differential effects on the ventricles in tachyarrhythmias.

In our study, pre-treatment with amiodarone reduced the occurrence of dissimilar rhythms and we only observed VF with similar rates. Amiodarone significantly increased the slope of the LV APD restitution curve, APD heterogeneity and it is likely this is linked to a greater propensity to developed VF. VF is often sustained by multiple activating wavefronts and is more chaotic in nature that VT (34, 35), and therefore propagating wavefront from the LV in VF are less likely to encounter periodically refractory tissue in the RV compared to a regular tachycardia such as VT. We found that amiodarone more regularly causes VF rather than VT after arrhythmia provocation, and no episodes of dissimilar rhythms were seen in the amiodarone group. Amiodarone has a complex mechanism of action as an antiarrhythmic, its pre-dominant mode of action involves prolonging the repolarization phase and APD through its inhibition of the outward potassium current. However, it also inhibits the inward sodium and calcium currents to a lesser degree, and causes non-competitive beta blockade (36). This prolongation of the refractory period and the APD with amiodarone in a conventional model of anatomical re-entry is unlikely to favor or stabilize VT. Thus, VF was the pre-dominant arrhythmia we recorded in our study with amiodarone pre-treatment.

### Interventricular Conduction Block and Differing Interventricular Activation Patterns Contribute to Dissimilar Ventricular Rhythms

When we optically mapped dissimilar VT rates, we observed that at the very short cycle lengths of activation, the LV APD continued to shorten whereas the RV reached a relative plateau. A functional line of conduction block developed between the ventricles. At very high VT rates it is probable that the LV is able to shorten its effective refractory period (ERP) to allow for conduction and for tachyarrhythmias to propagate. However, in the RV the ERP would have remained relatively fixed resulting in conduction block at high VT rates from the LV.

### Clinical Implications

Dissimilar interventricular rhythms may be an avoidable and unappreciated cause of death in patients with ICDs and cardiac resynchronization therapy defibrillator (CRT-D) devices. Routine implantation of an LV lead in the coronary sinus alongside an RV ICD lead should be considered. This would allow early detection and treatment of VT and VF in the LV in instances where the RV rate of the ventricular arrhythmia is slower and below the treatment zone. With CRT-Ds, programming the device to sense and deliver therapy based on the LV lead data in addition to RV lead can be easily achieved.

### Limitations

Optical recordings of transmembrane potential are limited to a depth of only a few cells at the subepicardium, as such there is no data on intramural and endocardial activation patterns or restitution. Deductions on mechanisms of dissimilar rhythms and APD restitution properties are thus extrapolated from epicardial activation only. Dissimilar interventricular VT rates and ventricular rhythms are rare, and in this study we were able to optically map the mechanism in only a few hearts. It is likely, where dissimilar interventricular VT rates and ventricular rhythms occur other more complex transmural mechanisms may exist.

## Conclusion

Whilst dissimilar ventricular rhythms may be clinically rarely recorded, in the rat model we have demonstrated dissimilar VT rates occurring in the left and right ventricles. Sodium channel blockade with lidocaine can lower the threshold at which dissimilar ventricular rhythms develop. The difference in action potential duration restitution between the LV and RV is a possible underlying mechanism of dissimilar interventricular rhythms.

## Data Availability

The datasets generated for this study are available on request to the corresponding author.

## Ethics Statement

This work was performed in accordance with standards set out in the United Kingdom Animals (Scientific Procedures) Act 1986 and was approved by Imperial College London Ethical Review Board.

## Author Contributions

BH conducted the experiments, analyzed and interpreted the data, and wrote the manuscript. SL conducted some of the initial experiments and data analysis. JC-G and XL provided and adapted the MATLAB codes for analysis of optical fluorescence data and phase analysis. CM aided with the initial optical mapping studies. IW provided and selected the clinical traces from intracardiac devices. RC and NSP provided expertise is data interpretation and statistical analysis. FSN conceived and designed the project, reviewed the manuscript, and provided overall supervision of the project.

### Conflict of Interest Statement

The authors declare that the research was conducted in the absence of any commercial or financial relationships that could be construed as a potential conflict of interest.

## Supplementary Material

Supplementary Data–Video 1 Phase maps of dissimilar ventricular rhythms (VT in RV and VF in LV).
